# Influence of Genetic Variants in TPMT and COMT Associated with Cisplatin Induced Hearing Loss in Patients with Cancer: Two New Cohorts and a Meta-Analysis Reveal Significant Heterogeneity between Cohorts

**DOI:** 10.1371/journal.pone.0115869

**Published:** 2014-12-31

**Authors:** Melanie M. Hagleitner, Marieke J. H. Coenen, Ana Patino-Garcia, Eveline S. J. M. de Bont, Anna Gonzalez-Neira, Hanneke I. Vos, Frank N. van Leeuwen, Hans Gelderblom, Peter M. Hoogerbrugge, Henk-Jan Guchelaar, Maroeska W. M. te Loo

**Affiliations:** 1 Department of Pediatric Hematology and Oncology, Radboud University Medical Center, Nijmegen, The Netherlands; 2 Department of Human Genetics, Radboud University Medical Center, Nijmegen, The Netherlands; 3 Department of Pediatrics, University of Navarra and University Clinic, Pamplona, Spain; 4 Department of Pediatric Hematology and Oncology, University Medical Center Groningen, University of Groningen, Groningen, The Netherlands; 5 Human Genotyping Unit-CeGen, Spanish National Cancer Research Center, Madrid, Spain; 6 Department of Clinical Oncology, Leiden University Medical Center, Leiden, The Netherlands; 7 Department of Clinical Pharmacy & Toxicology, Leiden University Medical Center, Leiden, The Netherlands; University of Central Greece, Greece

## Abstract

Treatment with cisplatin-containing chemotherapy regimens causes hearing loss in 40–60% of cancer patients. It has been suggested that genetic variants in the genes encoding thiopurine S-methyltransferase (*TPMT*) and catechol O-methyltransferase (*COMT*) can predict the development of cisplatin-induced ototoxicity and may explain interindividual variability in sensitivity to cisplatin-induced hearing loss. Two recently published studies however, sought to validate these findings and showed inconsistent results. The aim of this study was to evaluate the role of polymorphisms in the TPMT and COMT genes in cisplatin-induced ototoxicity. Therefore we investigated two independent cohorts of 110 Dutch and 38 Spanish patients with osteosarcoma and performed a meta-analysis including all previously published studies resulting in a total population of 664 patients with cancer. With this largest meta-analysis performed to date, we show that the influence of *TPMT* and *COMT* on the development of cisplatin-induced hearing loss may be less important than previously suggested.

## Introduction

Cisplatin is an effective chemotherapeutic drug for several types of cancer such as ovarian cancer, lung cancer, osteosarcoma and neuroblastoma. Ototoxicity, characterized as a permanent, bilateral sensorineural hearing loss, is one of the most common side effects of cisplatin and occurs in 40–60% of patients [1]. Ototoxicity is one of the main reasons for dose reduction or termination of treatment with cisplatin treatment. Clinical risk factors for the development of cisplatin-induced ototoxicity have been described such as age younger than 5 years at diagnosis [2], [3], concurrent medication like carboplatin [4] as well as higher cumulative doses [2], [4], cranial radiation [5] and pre-existing renal dysfunction. Nevertheless, these clinical factors are not sufficient to reliably predict ototoxicity before the start of treatment. A candidate gene study in 166 pediatric patients suggested that genetic variants in the genes encoding *thiopurine S-methyltransferase* (*TPMT*) and *catechol O-methyltransferase* (*COMT*) can predict the development of cisplatin-induced ototoxicity and may explain the interindividual variability [6]. Two recently published studies, developed to validate these findings, showed inconsistent results. Pussegoda and colleagues confirmed the findings of Ross et al. for variants in the *TPMT* gene in a cohort of 155 patients however, with smaller effect sizes compared to the original findings [7]. In contrast, no association of *TPMT* or *COMT* and ototoxicity was found in a cohort of 213 patients with medulloblastoma [8]. Based on these three studies no recommendation for clinical implementation regarding genetic variants in *TPMT* and *COMT* and ototoxicity can be made. The aim of the current study is to provide a clearer picture of the genetic impact of *TPMT* and *COMT* variants on developing cisplatin-induced ototoxicity. Therefore we investigated two independent cohorts of 110 Dutch and 38 Spanish patients with osteosarcoma and performed a meta-analysis including previously published studies [6]–[8] resulting in a total population of 664 patients with cancer.

## Materials and Methods

### Dutch cohort

A cohort of 110 Dutch patients with high-grade osteosarcoma treated with cisplatin was recruited from the Radboud University Medical Centre, the University Medical Centre of Groningen and Leiden University Medical Center. All patients were treated with cisplatin with a median cumulative dose of 500 mg/m^2^ (range: 100 to 600 mg/m^2^). Of patients included audiometric analysis were available at diagnose, during therapy and after completion of therapy. Data concerning administration of other potentially ototoxic medications such as furosemide, vancomycin, gentamicin, tobramycin, amphotericin B, carboplatin and vincristine, were recorded. In patients alive, DNA was extracted from blood using the QIAamp DNA Blood Midi kit (Qiagen Venlo, The Netherlands) or saliva (2 ml) using the Oragene DNA purification protocol (DNA Genotek, Kanata, Ontario, Canada). Of patients that passed away, (N = 39, 26.5%, median follow up time 2.3 years with range 1.1–5.5 years), DNA of paraffin-embedded samples was extracted as recently described [9]. The study was approved by the ethics committee from the Radboud University Medical Centre as the central committee for this study for the Netherlands and written informed consent was obtained from parents and/or patients

### Spanish cohort

To enlarge sample size for meta-analyses, we additionally included an independent Spanish cohort of osteosarcoma patients who were treated with cisplatin-containing regimens. To prevent population bias, this cohort was analyzed separately. In total, 38 patients with osteosarcoma were included on the basis of availability of germline DNA and audiologic assessment at least one month after initial treatment. Data regarding ototoxic medication were available. The study was approved by the local ethics committee from the University of Navarra and University Clinic in Pamplona. Written informed consent was obtained from parents and/or patients.

### Ototoxicity

In both cohorts audiologic evaluations were prospectively performed at diagnosis, during therapy and after completion of therapy. First audiological follow-up was performed 1–3 months after completion of therapy and thereafter annually. All audiometric assessments were age appropriate performed by conventional or play audiometry under standardized conditions as part of routine clinical monitoring for cisplatin-related hearing loss. Hearing loss was retrospectively classified according to two grading systems: the National Cancer Institute CTCAE version 3.0 (http://ctep.cancer.gov/forms/CTCAEv3.pdf) and the new SIOP Boston ototoxicity scale which classifies hearing loss on the basis of absolute hearing levels in 4 grades [10]. The most recent audiologic assessment during follow-up period after the last cisplatin course was used for analysis.

### Genotyping

Previously associated variants in *TPMT* (rs12201199: C__31923406_10, rs1800460: C__30634116_20, rs1142345: C_____19567_20) and *COMT* (rs4646316: C__29193982_10, rs9332377: C__29614343_10) were genotyped using Taqman allelic discrimination assays according to the protocol of the manufacturer (Invitrogen, Bleiswijk, The Netherlands). After amplification, the fluorescent signal for allelic discrimination was determined using the 7500 Fast Real-time System (Invitrogen). Automated allele calling was performed by allelic discrimination plots using SDS 1.4 software (Invitrogen).

### SNP analysis

In both cohorts, statistical differences regarding demographic data between patients who developed hearing loss (cases;>20 dB hearing loss above 4 kHz) and patients without hearing loss (controls; ≤20 dB hearing loss at all frequencies) were assessed by the Fischer exact test. Reported p-values are two-sided and are considered statistically significant if <0.05. Associations between ototoxicity and potential confounders, such as age, gender, cumulative dosage of cisplatin and concomitant drugs were evaluated using a 2×2 table (in case of dichotomized variables) or linear regression (in case of linear variables) in SPSS version 20 (SPSS Inc., Chicago, Il). To assess the effect of the genetic variants on ototoxicity data were dichotomized in grade 0 vs. grade 2–4. Multivariate logistic regression analyses included vincristine exposure as confounder in PLINK [11] using the command –logistic.

### Meta-analyses

We searched PubMed for papers using the keywords: ‘ototoxicity’, ‘TPMT’ and ‘COMT’. Since the publication of Ross and colleagues [6] in 2009 only two other studies [7], [8] evaluated the association of the *COMT* and *TPMT* variants in cancer patients. Meta-analysis was performed on these three previously published studies and the two cohorts presented in this study were included. Of each of the five studies the CTCAE hearing loss criteria were used, excluding patients with grade 1 ototoxicity to better differentiate between cisplatin-induced ototoxicity and normal hearing. The studies were weighed using the inverse variance method, where larger studies with smaller standard errors have more weight than smaller studies with larger errors (Review manager 5.0, The Cochrane Collaboration, Oxford, UK). Heterogeneity among studies was examined with I^2^ statistics that can be interpreted as the proportion of total variation contributed by between-study variation. Allelic odds ratio (OR) were recalculated dependent on the number of cases and controls in each cohort and 95% confidence interval (CI) were estimated using a fixed effects model or random effects models in case large heterogeneity I^2^>50% was observed.

## Results

### Dutch cohort

A total of 110 patients with Dutch ancestry and newly diagnosed osteosarcoma were included of whom 42 (38.2%) developed>20 dB hearing loss above 4 kHz. Characteristics of patients divided in patients with hearing loss (cases;>20 dB hearing loss above 4 kHz) and patients without hearing loss (controls; ≤20 dB hearing loss at all frequencies) are depicted in **Table 1**. Baseline characteristics did not show statistically significant differences between the two groups. None of the patients included was treated with cranial irradiation or with concomitant ototoxic antibiotics. Only five patients were additionally treated with vincristine. Other ototoxic chemotherapeutics were not administered. In 23 (20.9%) of all patients cumulative dose of cisplatin was reduced due to ototoxicity (N = 9, >20 dB hearing loss above 4 kHz) or other cisplatin-induced toxicity (N = 14). Baseline audiograms were available in 87 patients (79%) showing normal baseline hearing thresholds (≤20 dB) in all patients. Median follow up period was 5.2 years (range 23–7763 days). Different ototoxicity grading systems were used to exclude biased dichotomisation. For the CTCAE criteria, 80 patients with baseline audiogram were included. Patients with grade 1 ototoxicity (N = 7) were excluded in order to use same inclusion/exclusion criteria as previous studies in literature [6]–[8]. Classification according to the CTCAE criteria showed in all but seven patients identical toxicity grades when compared to the SIOP grading system. Four patients with grade 1 and three patients with grade 2 ototoxicity according to the SIOP Boston ototoxicity scale were upgraded to grade 2 and 3 according the CTCAE criteria. Hearing loss grade 2–4 was seen in 23 patients graded according the CTCAE criteria (0 vs.>25 dB at 4–8 kHz) and 22 according to the SIOP criteria (0 vs.>20 dB at 4–8 kHz). Univariate analysis showed no association with non-genetic factors previously reported to be associated with ototoxicity: age (p = 0.73) and cumulative dose of cisplatin (p = 0.99).

**Table 1:** Demographic data of the Dutch and Spanish cohort.

	Dutch cohort			Spanish cohort		
	Cases	Controls		Cases	Controls	
	= Ototoxicity	= No ototoxicity		= Ototoxicity	= No ototoxicity	
	N = 42	N = 68	*p*-value	N = 16	N = 22	*p-*value
**Age at diagnosis** (y)	15	15	0.82	11.5	14	0.23
median, range	(5–40)	(7–39.3)		(4–29)	(7–28)	
**Gender** (male)	22	33	0.6	15	6	0.1
N, %	(40%)	(60%)		(71.4%)	(28.6%)	
**Cumulative dose** **cisplatin** (mg/m2)	500	480	1	504	515	0.32
median, range	(100–600)	(200–600)		(120–870)	(140–720)	
**Concomitant drugs**						
Vincristine (N, %)	3 (2.7%)	2 (1.8%)	0.3	2 (5.2%)	4 (10.5%)	0.13
Aminoglyocide antibiotics (N, %)	0	0		15 (68.2%)	10 (62.5%)	0.74
Otoprotectants (N, %)	0	0		0	0	

### Spanish cohort

To enlarge sample size for the meta-analyses we included another cohort of 38 Spanish patients of European ancestry with osteosarcoma (**Table 1**). No statistically significant differences concerning age, gender or cumulative dose of cisplatin was seen between patients with hearing loss (cases; >20 dB hearing loss above 4 kHz) and patients with normal hearing (controls; ≤20 dB hearing loss at all frequencies). None of the included patients was additionally treated with cranial irradiation. Regarding concomitant medication, all but six patients were additionally treated with vincristine and 23 patients received ototoxic antibiotics, such as vancomycin, gentamicin or tobramycin. In univariate analysis the use of these antibiotics was not a confounding factor to develop ototoxicity in this cohort (p = 0.74). As to the small sample size and to prevent population bias due to ethnicity, genotyping results of this cohort were analyzed as an independent cohort in the meta-analyses.

### Genotyping

We genotyped three variants in the *TPMT* gene (rs12201199, rs1800460, and rs1142345) and two in the *COMT* gene (rs4646316, rs9332377). No deviation from Hardy–Weinberg equilibrium was observed. Irrespective of the grading system used (CTCAE or SIOP), association analysis using an additive genetic model correcting for usage of vincristine, showed no association between genetic variants in *TPMT* or *COMT* with an increased risk of ototoxicity in the Dutch cohort (**S1 Table** **,** **S2Table** **and** **S3 Table**).

### Meta-analyses

**Figure 1:**
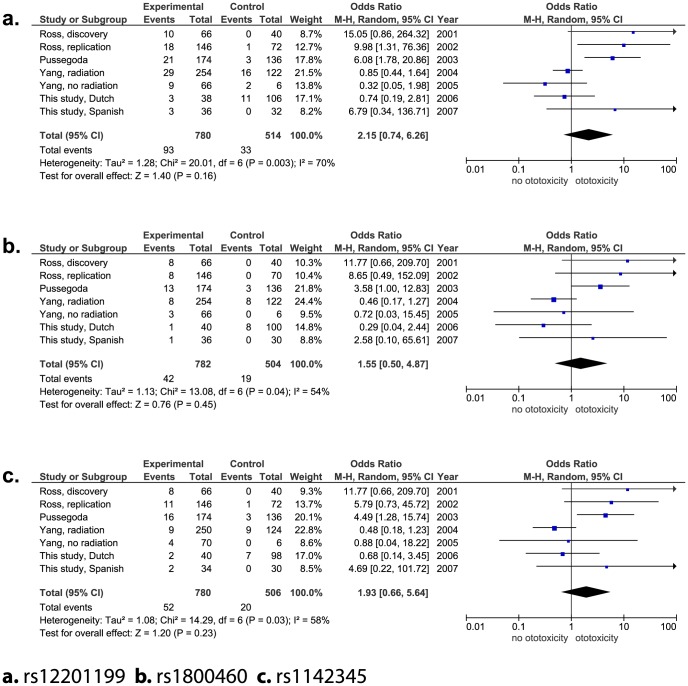
Forest plots genetic variants in *TPMT* gene.

**Figure 2:**
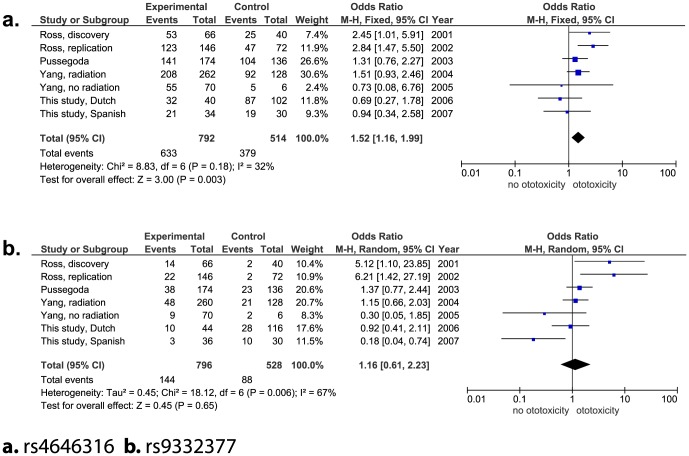
Forest plots genetic variants in *COMT* gene.

So far, three studies with four independent cohorts studying the influence of *TPMT* and *COMT* on the development of ototoxicity have been published [6]–[8]. With this study we add two additional independent cohorts. In all studies the CTCAE criteria were reported. To better differentiate between cases and controls all studies excluded patients with grade 1 ototoxicity. In total, 664 patients were included in the meta-analyses. **Table 2** shows the demographic data of the initial studies, as well as the number of patients per study that were included in the meta-analysis. In **Figs. 1** and **2** odds ratios for each individual study are presented. The genetic variant rs4646316 in the *COMT* gene was the only significant association with ototoxicity (OR A versus T allele: 1.52, 95% CI: 1.16–1.99, P = 0.003; **Fig. 2**). The heterogeneity test showed a non-statistically significant heterogeneity with an I^2^ of 31%. Notably, there are substantial differences in the range of the “total events” captured between the meta-analyses which is related to incomplete genotyping in all studies, except for the study of Pussegoda and colleagues [7].

**Table 2:** Demographic data of patients with ototoxicity included for meta-analysis.

	Cases/Total	Ethnicity	Age (y)	Types of cancer	Gender of cases	Cumulative dose	Cranial irradiation	Otoprotectant	Vincristine	Aminoglycocide
	*N*		*Median*		*Male %*	*Median* (mg/m^2^)	*%*		used	antibiotics
Ross et al.^6^	106/162	Mixed population	6	Different cancers	67.0%	400 mg/m^2^	18.50%	No information	Yes	Yes
Pussegoda et al.^7^	87/155	Mixed population	6	Different cancers	49.4%	400 mg/m^2^	18%	No information	Yes	Yes
Yang et al., radiation cohort^8^	131/195	Mixed population	7	Medulloblastoma	68.8%*	300 mg/m^2^*	100%*	Yes	Yes	No information
Yang et al., no radiation^8^	35/38	Mixed population	8	Medulloblastoma	65.0%	500 mg/m^2^	0	Yes	Yes	No information
Hagleitner, this study	22/80	Dutch ancestry	15	Osteosarcoma	40.0%	500 mg/m^2^	0	No	Yes	No
Patino-Garcia, this study	18/34	European ancestry	12	Osteosarcoma	55.3%	500 mg/m^2^	0	No	Yes	Yes
*data of total group as published by Yang et al.										

## Discussion

Although, the results of Ross and colleagues investigating the influence of variants in *TPMT* and *COMT* were very promising [6], the outcome of more recent studies argues against the premise that these variants might help to identify patients at risk for ototoxicity. In the present study, therefore, we aimed to gain a clearer picture on the role of these variants by adding additional data on the subjects, and by performing a meta-analysis including two new cohorts.

Our Dutch dataset of 110 uniformly treated patients with osteosarcoma, is a homogenous group without potentially confounding factors such as cranial radiation and concomitant medication. For classification of cisplatin-induced hearing loss, we preferred to use the SIOP Boston scale as it combines the two main types of measuring ototoxicity, namely changes of hearing from baseline as well as absolute hearing levels [10]. As previously published studies used the CTCAE criteria for the classification of ototoxicity, we reclassified our data according to the CTCAE criteria, and excluded grade 1 ototoxicity to better distinguish between cases and controls. In the Dutch cohort, and also the other newly analyzed Spanish cohort, we did not observe any statistically significant association between the genetic variants in *TPMT*, *COMT* and ototoxicity. We did observe differences between the two cohorts such as lower ototoxicity rate in the Dutch cohort which might be explained by the inclusion of adults until the age of 40 years. It is known that young children are more susceptible to ototoxicity from cisplatin [3]. Furthermore, the Dutch cohort included patients who have already died. It might be that controls that died have become cases if they have survived longer. However, the median follow up time of these patients who have died was 2.3 years with a range between 1.1 and 5.5 years. Studies included in this meta-analysis showed universally that the majority of ototoxicity occurred between 0.5 and 6 months from start of cisplatin chemotherapy [6]–[8].

After meta-analyses of the genetic variants in *TPMT* and *COMT* including in total 664 patients only rs4646316 variant in the *COMT* gene remained statistically significant. However, the effect was much smaller than reported in previously published studies. In the initial discovery cohort of Ross and colleagues the OR of this variant to develop ototoxicity was 2.5 (95% CI: 1.5–4.3) [6]. Whereas the combined effect of all studies included in the meta-analysis showed an OR of 1.52 (95% CI: 1.16–1.99). Three of the cohorts (excluding the initial study by Ross et al.) showed the same direction of effect for rs4646316, however, this was not statistically significant. The *COMT* gene encodes for the COMT enzyme which is involved in the inactivation of catecholamine neurotransmitters. Although, it has been shown that it is also highly expressed in sensory hair cells of the inner ear, its role regarding auditory function remains unclear [12]. In addition, the influence of the variant rs4646361 on the COMT enzyme has not been studied yet.

Generally, the association between *TPMT* variants and cisplatin ototoxicity was unexpected [9], as it was not previously linked to cisplatin metabolism. Recent published data support the hypothesis that *TPMT* might have an influence on cisplatin metabolism by increasing levels of S-adenosylmethionine (SAM) [13]. Furthermore, a recent study demonstrated that functional *TPMT* variants were associated with progression-free survival in cisplatin-based chemotherapy outcomes in ovarian cancer [14]. However, in vivo studies in mice with different *TPMT* genotypes showed no differences in hearing damage between TPMT wild-type and knockout mice [8].

With the largest meta-analysis performed to date we show that the influence of *TPMT* and *COMT* on the development of cisplatin-induced hearing loss may be less pronounced than previously suggested. This lack of association observed might be attributable to heterogeneity between the different studies as shown in Table 2. First, different patient populations with different ethnicities were used in the studies which could account for the differences in the genetic associations in the meta-analysis. Second, in the study cohorts with clear positive association, a heterogeneous group of patients with respectively 9 and 12 different types of cancers was included [6], [7]. As a consequence, patients were treated with different treatment regimens sometimes including cranial irradiation and different co-medication. In these studies an analysis for specific patient subgroups was not performed probably due to the limited number of patients included for each subgroup. Also the cohorts with clear negative association included patients with different cancer types, one study included patients with osteosarcoma (this study) as in the study of Yang et al. patients with medulloblastoma have been included which necessitates highly different treatment protocols regarding cumulative dose of cisplatin, cranial radiation and concomitant medication. Third, the different types of cancer were not equally distributed among patients with and without ototoxicity, for example in the cohort of solid tumors investigated by Yang and colleagues 35 of the 38 patients [8] were reported to have hearing loss which may lead to selection bias. Fourth, the median cumulative doses of cisplatin ranged from 300 to 500 mg/m^2^ in the different studies. However, neither in the study cohort of medulloblastomas with a relatively low cumulative dose of cisplatin [8] nor in osteosarcoma patients treated with higher doses of cisplatin (this study), an association was found. In addition, young age has recently been pointed out as an important factor to develop cisplatin-induced hearing loss [3], which may be the reason for the lower incidence of ototoxicity in the osteosarcoma cohort as the median age at diagnosis is older compared to the other cohorts. At last, the use of otoprotective agents, such as amifostine, may be a potentially confounding factor. A Cochrane study on this topic was due to methodological limitations of individual studies unable to show evidence of effect to use amifostine as an otoprotective intervention [15]. However, there was also no evidence that it had no effect as otoprotectant. In the study of Yang et al the use of amifostine was linked to less ototoxicity [8].

It is clear that all studies included in this meta-analysis have confounding variables which could potentially influence the development of ototoxicity. This is important given the inverse method of weighing studies which has been used in this meta-analysis. The studies of Pussegoda et al and Yang et al were consistently attributed the highest weighting. Pussegoda et al included a diverse population of different cancers with different treatment protocols, the youngest population in this meta-analysis with different follow-up time between cases (5 years follow-up) and controls (2 years follow-up). The radiation cohort of Yang et al patients received highly ototoxic cranial irradiation, but 90% also received an otoprotectant, and overall these patients received a significantly lower cumulative dose of cisplatin. Thus, the weight attributed to a study may affect the results of the meta-analysis, however this is something that cannot be circumvented in this type of analysis.

Nevertheless, younger age and higher cumulative doses of cisplatin appear to be the most consistent risk factors for ototoxicity. However, an upfront risk stratification based on these two factors is unreliable as these factors seem to be protocol specific and dose adjustments may jeopardize treatment efficacy of cisplatin. The addition of otoprotective agents in combination with cisplatin might be an alternative treatment strategy to prevent hearing loss. Although, the mechanism of cisplatin-induced hearing loss is not fully understood, several preclinical studies have shown that in the cochlear cells cisplatin induces the production of toxic levels of reactive oxygen species (ROS) which can initiate cochlear cell death leading to hearing loss [16], [17]. Numerous studies have investigated a variety of antioxidant agents to protect cochlea cells from cisplatin damage. Among them, sodium thiosulfate, D or L-methionine, glutathione ester and amifostine were successfully tested in animal models [18], [19]. The efficacy of otoprotective agents in patients is however disappointing. For instance, sodium thiosulfate showed an unwanted compromised antitumor effect of cisplatin [20] and no otoprotection was observed in children with germ cell tumors treated with amifostine in combination with cisplatin [21].

It remains challenging to find the right balance to maintain antitumor effect of cisplatin and on the other hand to prevent unwanted adverse effects. Innovative studies translating basic science into clinical practice are needed to unravel both, the mechanism of cisplatin-induced hearing loss and identification of patients at risk to develop ototoxicity. Pharmacogenetics may help to find new target genes, unfortunately it seems that the genes *TPMT* and *COMT* play only a minor role in cisplatin-induced ototoxicity and should therefore not guide clinical decision making for cisplatin dosing.

## Supporting Information

S1 Table Minor allele frequency of Dutch cohort.
(DOCX)

S2 Table Association analyses of Dutch cohort.
(DOCX)

S3 Table Call rates of the Dutch and Spanish cohort for each variant according to the CTCAE ototoxicity criteria.
(DOCX)

S1 Checklist (DOC)
