# Supplementary material for: Influence of Genetic Variants in TPMT and COMT Associated with Cisplatin Induced Hearing Loss in Patients with Cancer: Two New Cohorts and a Meta-Analysis Reveal Significant Heterogeneity between Cohorts
Source: PLoS One. 2014 Dec 31;9(12):e115869. doi: 10.1371/journal.pone.0115869 (PMC4281251; doi:10.1371/journal.pone.0115869)
Supplement: S1 Table — Minor allele frequency of Dutch cohort. (DOCX) [file pone.0115869.s001.docx]

| Gene | SNP | minor allele | MAF |
| --- | --- | --- | --- |
| *TPMT* | rs1142345 | G | 6.5% |
|  | rs1800460 | T | 6.4% |
|  | rs12201199 | T | 9.7% |
| *COMT* | rs4646316 | A | 16.2% |
|  | rs9332377 | T | 23.7% |
| MAF=minor allele frequency | | |  |

**Supplementary Table 1.** Minor allele frequency of Dutch cohort
