# Supplementary material for: Influence of Genetic Variants in TPMT and COMT Associated with Cisplatin Induced Hearing Loss in Patients with Cancer: Two New Cohorts and a Meta-Analysis Reveal Significant Heterogeneity between Cohorts
Source: PLoS One. 2014 Dec 31;9(12):e115869. doi: 10.1371/journal.pone.0115869 (PMC4281251; doi:10.1371/journal.pone.0115869)
Supplement: S2 Table — Association analyses of Dutch cohort. (DOCX) [file pone.0115869.s002.docx]

| **Supplementary Table 2.** Association analyses of the Dutch cohort (SIOP vs. CTCAE criteria) | | | | | | | | | | | | | | | | | | | | |
| --- | --- | --- | --- | --- | --- | --- | --- | --- | --- | --- | --- | --- | --- | --- | --- | --- | --- | --- | --- | --- |
|  |  |  | |  | | | |  |  | |  | |  | |  |  | |  |  |  |
|  |  | **SIOP Boston criteria** | | | | | | **CTCAE criteria** | | | | | | |  |  | | | | |
| Gene | SNP | OR | 95% CI | | | *p*-value |  | OR | | 95% CI | | | | *p*-value | | |  |  |  |  |
| *TPMT* | rs1142345 | 0.96 | 0.30 | | 3.08 | 0.95 |  | 0.71 | | 0.15 | | 3.26 | | 0.66 | | |  |  |  |  |
|  | rs1800460 | 0.49 | 0.12 | | 1.97 | 0.31 |  | 0.32 | | 0.04 | | 2.58 | | 0.28 | | |  |  |  |  |
|  | rs12201199 | 0.65 | 0.22 | | 1.91 | 0.44 |  | 0.74 | | 0.20 | | 2.76 | | 0.65 | | |  |  |  |  |
| *COMT* | rs4646316 | 0.49 | 0.22 | | 1.14 | 0.10 |  | 0.67 | | 1.81 | | 0.24 | | 0.42 | | |  |  |  |  |
|  | rs9332377 | 0.80 | 0.41 | | 1.55 | 0.51 |  | 0.91 | | 0.41 | | 2.04 | | 0.83 | | |  |  |  |  |
|  | |  | |  | | | |  |  | |  | |  | |  |  |  |  |  |  |
