# Supplementary material for: Influence of Genetic Variants in TPMT and COMT Associated with Cisplatin Induced Hearing Loss in Patients with Cancer: Two New Cohorts and a Meta-Analysis Reveal Significant Heterogeneity between Cohorts
Source: PLoS One. 2014 Dec 31;9(12):e115869. doi: 10.1371/journal.pone.0115869 (PMC4281251; doi:10.1371/journal.pone.0115869)
Supplement: S3 Table — Call rates of the Dutch and Spanish cohort for each variant according to the CTCAE ototoxicity criteria. (DOCX) [file pone.0115869.s003.docx]

| **Supplementary Table 3.** Call rates of the Dutch and Spanish cohort for each variant according to the CTCAE ototoxicity criteria. | | | | | | |  |  |  |  |  |
| --- | --- | --- | --- | --- | --- | --- | --- | --- | --- | --- | --- |
|  |  |  |  |  |  | | |  |  |  |  |
| Gene | SNP | **Dutch Cohort, N=80** |  | **Spanish Cohort, N=34** | |  | |  |  |  |  |
| *TPMT* | rs1142345 | 69 |  | 32 |  | | |  |  |  |  |
|  | rs1800460 | 70 |  | 33 |  | | |  |  |  |  |
|  | rs12201199 | 72 |  | 34 |  | | |  |  |  |  |
| *COMT* | rs4646316 | 71 |  | 32 |  | | |  |  |  |  |
|  | rs9332377 | 80 |  | 33 |  | | |  |  |  |  |
